# Triptolide: pharmacological spectrum, biosynthesis, chemical synthesis and derivatives

**DOI:** 10.7150/thno.57745

**Published:** 2021-05-24

**Authors:** Jie Gao, Yifeng Zhang, Xihong Liu, Xiayi Wu, Luqi Huang, Wei Gao

**Affiliations:** 1 School of Traditional Chinese Medicine, Capital Medical University, Beijing, 100069, China.; 2 Beijing Shijitan Hospital, Capital Medical University, Beijing, 100038, China.; 3 State Key Laboratory Breeding Base of Dao-di Herbs, National Resource Center for Chinese Materia Medica, Chinese Academy of Chinese Medical Sciences, Beijing, 100700, China.; 4 Basic Medical College, Henan University of Chinese Medicine, Zhengzhou 450046, China.; 5 Advanced Innovation Center for Human Brain Protection, Capital Medical University, Beijing, 100069, China.

**Keywords:** *Tripterygium wilfordii*, Triptolide, Biological activities, Biosynthesis, Chemical synthesis

## Abstract

Triptolide, an abietane-type diterpenoid isolated from* Tripterygium wilfordii* Hook. F., has significant pharmacological activity. Research results show that triptolide has obvious inhibitory effects on many solid tumors. Therefore, triptolide has become one of the lead compounds candidates for being the next "blockbuster" drug, and multiple triptolide derivatives have entered clinical research. An increasing number of researchers have developed triptolide synthesis methods to meet the clinical need. To provide new ideas for researchers in different disciplines and connect different disciplines with researchers aiming to solve scientific problems more efficiently, this article reviews the research progress made with analyzes of triptolide pharmacological activity, biosynthetic pathways, and chemical synthesis pathways and reported in toxicological and clinical studies of derivatives over the past 20 years, which have laid the foundation for subsequent researchers to study triptolide in many ways.

## 1. Introduction

**Figure 1:**
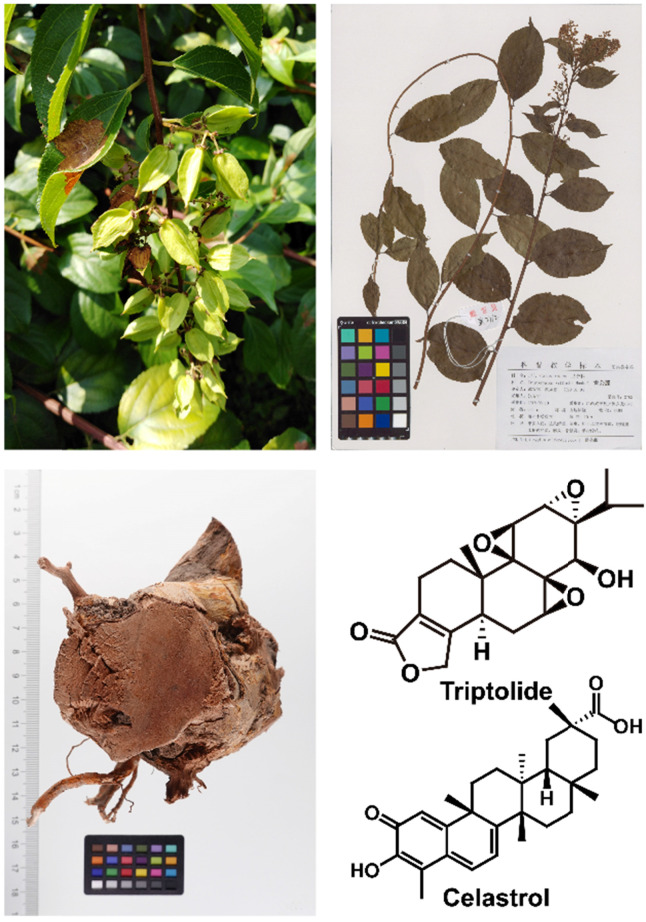
*Tripterygium wilfordii* and its important active ingredients.

**Figure 2:**
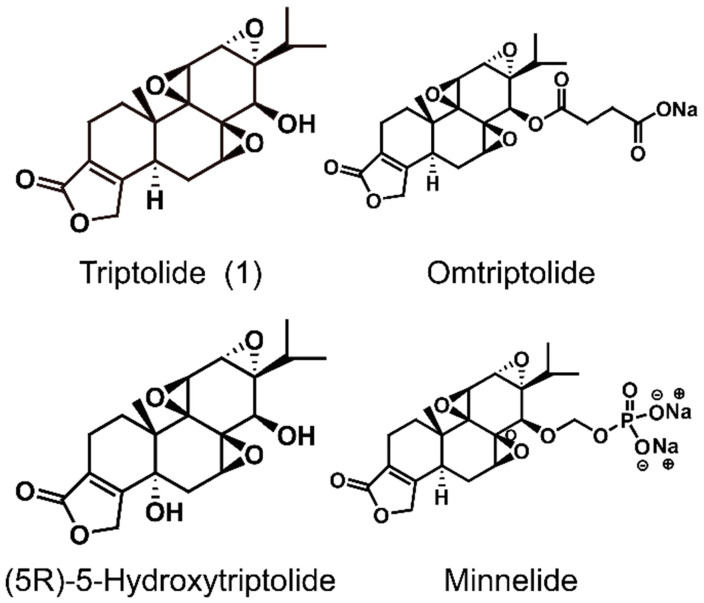
The structures of triptolide (**1**) and its derivatives.

*Tripterygium wilfordii* Hook. F. is a traditional Chinese medicinal herb, commonly known as "Thunder God Vine" or "Lei Gong Teng" (Figure 1). It is mainly used to treat autoimmune and inflammatory diseases, such as rheumatoid arthritis and systemic lupus erythematosus 1, and its main active compounds are terpenoids including triptolide and celastrol. Triptolide (Figure 2) is currently considered to be one of the active compounds most likely to translate from traditional to modern medicine 2.

**Figure 3:**
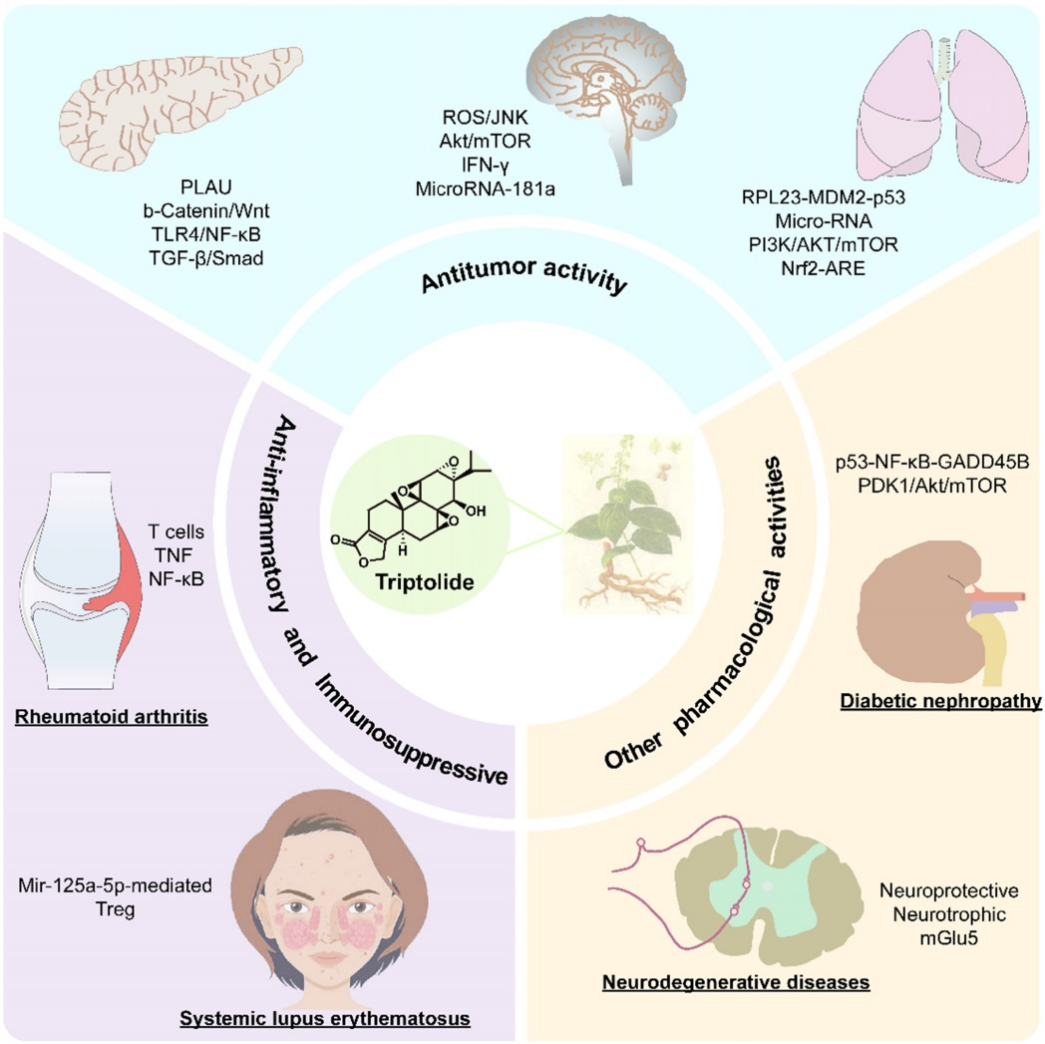
The pharmacological activity and mechanism of triptolide.

Triptolide is an important diterpene active compound in *T. wilfordii*. Since 1972, when Kupchan et al. first isolated triptolide and found its significant anti-leukaemic effects 3, it has also been proven to have significant anti-inflammatory, immunosuppressive, anticancer and other important biological activities (Figure 3) 4. Currently, triptolide is mainly obtained from its extraction from medicinal plants and separation from other compounds. Due to the extremely low content of triptolide in medicinal plants (~66.5 μg/g) 5, chemical extraction and separation are insufficient to meet the needs of industrialization. Triptolide is an abietane-type diterpene with 3 epoxy groups and an α, β-unsaturated five-membered lactone ring structure, which makes the chemical synthesis process challenging and not commercially viable on an industrial scale. Therefore, the current research focus is the biosynthesis of triptolide and its precursor. In recent years, with increasingly intensive study into traditional Chinese medicine (TCM), researchers have developed medications based on active compounds such as artemisinin, Taxol and other effective compounds used in TCM. Moreover, artemisinin and paclitaxel are also successful examples of using the principles of synthetic biology used to produce natural products or their precursor compounds at high yields.

In addition, an increasing number of scientific research problems can be solved by interdisciplinary contributions. For example, predicting protein folding structure through AI technology considered among the top ten scientific breakthroughs of *science*. This example provides a reference for scientific researchers seeking breakthroughs of technical bottlenecks. By combining the ideas used in different disciplines to study triptolide, researchers may generate additional novel ideas.

Therefore, to obtain a deeper understanding of triptolide through the combination of multiple disciplinary approaches, we analyzed its biosynthetic pathway. Triptolide and its precursors were efficiently synthesized using the principles of synthetic biology, which laid the foundation for pharmacological research on triptolide, the precursor compounds used in triptolide biosynthesis and triptolide derivatives. This article reviews the research progress on triptolide in terms of its pharmacological activity, biosynthesis, chemical synthesis, and toxicology and discusses recent clinical trials of its derivatives. This review will help researchers better understand all aspects of triptolide and provides constructive suggestions for the further study of triptolide.

## 2. Biological functions

### 2.1. Anti-inflammatory and immunosuppressive effects of triptolide

As a TCM herb, *T. wilfordii* has long been used to treat conditions characterized by rheumatism, including rheumatoid arthritis, nephritis and systemic lupus erythematosus. Its main effective component, triptolide, has obvious anti-inflammatory and immunosuppressive effects 1. Recent studies have shown that triptolide has a positive therapeutic effect on a variety of autoimmune and inflammatory diseases. It not only can induce apoptosis by inhibiting the proliferation of immune cells and inflammation-related cells but can also reduce the release of cytokines and pro-inflammatory mediators, thus inducing anti-inflammatory and immunosuppressive effects 4.

#### 2.1.1. Rheumatoid arthritis

Rheumatoid arthritis (RA) is an inflammatory, autoimmune disease. Multiple studies have shown that triptolide can be effectively used to treat RA through various mechanisms. These findings suggest that triptolide is one of the main compounds critical for the therapeutic effect of traditional Chinese herbal remedies on RA. The current research on the mechanism of RA treatment with triptolide mainly includes the following aspects: (1) Reduction in joint inflammation in RA by inhibiting T cell secretion of inflammatory cytokines 6, (2) amelioration of inflammation in RA by inhibiting angiogenesis at the sites of inflammation 7, (3) induction of fibroblast apoptosis to inhibit the inflammatory response in RA 8, (4) reduction in the degree of inflammation by inhibiting multiple signaling pathways (e.g., the TREM1 signaling pathway) 9 , (5) induction of OCP cell apoptosis by mediating the degradation of clap2, inhibiting OC formation, which has a therapeutic effect on RA 10.

To study the mechanisms by which triptolide exerts its effects in the treatment of rheumatoid arthritis, network pharmacology and molecular docking were used. Network pharmacology is a new discipline based on the theory of system biology, which analyzes the network of biological system and selects specific signal nodes for multi-target drug molecular design. Molecular docking is a method of drug design based on the characteristics of receptors and the interaction between receptors and drug molecules. First, considering network pharmacology, Yunbin Jiang et al. analyzed the anti-RA active compounds in *T. wilfordii* and concluded that triptolide and celastrol are the key active compounds. The data confirmed that the key molecular mechanism is related to the inhibition of the inflammatory response by inactivating the TNF and NF-κB signaling pathways 11. Xinqiang Song et al. organized the genes and proteins related to RA in public databases through a creative approach, interpretative phenomenological analysis (IPA). Subsequently, molecular docking was used to predict the binding pockets of the six top candidate triptolide target proteins: CD274, RELA, MCL1, MAPK8, CXCL8 and STAT1 12. However, network pharmacology is mainly used to analyze big data for predicting potential genes, targets, proteins or signaling pathways. This approach can only provide a certain degree of referent information for the treatment of RA with triptolide. Therefore, researchers need to be cautious and rigorous in the analysis of network pharmacology results.

Triptolide has a significant therapeutic effect on RA, but due to the own toxicity it induces, the current research hotspot involves technology using nanomaterials to carry triptolide to target the release to the lesion. Studies have shown that the use of poly-γ-glutamic acid-grafted di-tert-butyl L-aspartate hydrochloride (PAT) to prepare a TP-containing nanodrug carrier system can reduce the toxicity of triptolide ensuring the therapeutic effect of triptolide and revealing its potential as an effective drug candidate for RA 13. The use of amphiphilic pH-sensitive galactosyl dextran-retinal (GDR) nanoparticles to encapsulate triptolide may enhance the anti-inflammatory effect of CIA mouse models 14. The latest results confirmed that by encapsulating triptolide in the star-shaped amphiphilic block copolymer POSS-PCL-b-PDMAEMA, the constructed pH-sensitive triptolide nanomedicine can achieve significant anti-inflammatory effects at ultra-low doses to treat RA 15. The use of nanomaterials to carry triptolide has many advantages, such as targeted drug delivery and reduced triptolide dose. Nanomaterials provide effective solutions for accessing the narrow treatment window of triptolide. Nanomaterial carriers are examples of the combination of material chemistry and natural drugs, which in this case was used to address the limitations of triptolide.

#### 2.1.2. Systemic lupus erythematosus

Systemic lupus erythematosus (SLE) is a chronic autoimmune inflammatory disease. *T. wilfordii* has been used in the treatment of SLE for centuries, and has achieved remarkable results. Modern research shows that triptolide can alleviate SLE through miR-125a-5p-mediated upregulation of the Treg ratio 16.

#### 2.1.3 Mechanisms of the anti-inflammatory and immunosuppressive effects of triptolide

Research indicates that triptolide exerts its anti-inflammatory and immunosuppressive effects via multiple targets. First, triptolide can regulate signaling pathways, such as by inhibiting the nuclear factor-κB (NF-κB) signaling pathway 17, or inhibiting the IL-6/signal transducer and transcription 3 (STAT3)-activated signaling pathway and downregulating IL-17 18. Second, triptolide can inhibit the expression of pro-inflammatory molecules, such as VCAM-1, TGF-β, C3 and CD40 19, 20. In addition, it can also inhibit myofibroblast infiltration, thereby improving multiple organ fibrosis 21. With the continuous development of technology, researchers are clarifying the anti-inflammatory and immunosuppressive mechanisms of triptolide at the cellular, molecular and genetic levels.

### 2.2. Antitumor activity

Triptolide has significant, broad-spectrum antitumor and sensitizing effects. In recent years, increasing evidence has shown that triptolide has significant antitumor activity, with potential therapeutic effects on lung cancer, liver cancer, pancreatic cancer and other tumors 22, 23 (Table 1). The antitumor activity of triptolide is reflected in its strong cytotoxicity, which can nullify drug resistance, and inhibit neovascularization and tumor metastasis. Triptolide primarily exerts anticancer effects by inducing apoptosis 4. In recent years, studies have shown that autophagy and cell senescence are also involved in this process 24, 25. Triptolide is effective not only in ordinary tumor cells, but also in multidrug resistant cancer cells and some tumor stem cells 26, 27.

#### 2.2.1. Lung cancer

Lung cancer is a malignancy with some of the highest mortality rates in the world. Studies have shown that triptolide can regulate the ribosomal RPL23-MDM2-p53 signaling pathway to disintegrate the nucleolus and inhibit rRNA synthesis, ultimately inducing cell cycle arrest and apoptosis to inhibit cell proliferation and tumor growth 28. Reno et al. confirmed that triptolide can change the expression profile of miRNAs in lung cancer cells and inhibit the migration, invasion and metastasis of cancer cells 29. This research has provided new ideas for the treatment of lung cancer and confirmed that triptolide can be used as a potential lung cancer treatment drug.

Studies have shown that triptolide has a potential therapeutic effect on non-small cell lung cancer (NSCLC). It can induce NSCLC cell apoptosis; downregulate Akt, mTOR and P70S6K phosphorylation levels 30. At the same time, some researchers found that triptolide can reduce the Wnt signaling pathway, thereby reducing the proliferation of lung cancer cells, tumor formation and metastasis, to treat NSCLC. 31. In addition to its anticancer effect on NSCLC, triptolide can also target the Nrf2 pathway to reduce the chemotherapy resistance of cancer cells, which provides a new potential therapeutic strategy for NSCLC 32.

At this stage, the combination of triptolide was a hot issue concerning researchers. In one regimen, triptolide is combined with the low-dose anti-inflammatory drug aspirin to prevent lung cancer. Studies have shown that triptolide can activate p53 and inhibit NF-κB at the same time, which has the potential to treat human cancer, and aspirin can improve the efficacy of triptolide 33. The combination of anticancer drugs and anti-inflammatory drugs may be a promising method for the prevention and treatment of inflammation related cancers (such as lung cancer). In another combination of anticancer drugs, researchers designed lipid-polymer hybrid nanoparticles to serve as a coadministration system. Through *in vivo* and *in vitro* experiments, it was confirmed that the two drugs paclitaxel and triptolide in combination with LPN carriers had a synergistic effect in lung cancer transplantation and exhibited few systemic side effects 34. There are obvious differences between the two methods. One way is to improve the efficacy of anticancer drugs by inhibiting the pathological process of the cancer response. Another way is to combine different anticancer drugs to form a new drug delivery system, improve the synergy of drugs, and reduce the side effects of drugs and drug resistance.

#### 2.2.2. Liver cancer

In hepatocellular carcinoma (HCC), triptolide can inhibit the expression of miR-17-92 and miR-106b-25 by inhibiting c-Myc and upregulating their common target genes, thus leading to the death of HCC cells 35. Moreover, at different concentrations, triptolide was found to induce the phosphorylation of p53 at the serine-15 residue in HepG2 cells. Activating the tumor suppressor gene p53 can induce the apoptosis of liver cancer cells 36.

Nanomaterial preparation for use with triptolide have consistently been the research focus of scholars. In recent years, researchers have designed galactosylated chitosan TP-nanoparticles (GC-TP-NPs) to induce tumor cell apoptosis by blocking the TNF/NF-κB/BCL2 signaling pathway 23. These are promising drug candidates that prevent the progression of liver cancer while minimizing systemic toxicity.

#### 2.2.3. Neural tumors

Gliomas are common and lethal malignant primary brain tumors that exhibit strong invasion, rapid progression and susceptibility to relapse, leading to a poor prognosis for patients. It has been proven that triptolide not only can inhibit the proliferation of glioma cells and block the cell cycle in the G2/M phase but can also induce apoptosis and protective autophagy. Moreover, triptolide-induced apoptosis and autophagy of glioma cells can inhibit each other. Triptolide can regulate the cell cycle, apoptosis and autophagy by activating ROS / JNK inhibitory functions and the Akt / mTOR signaling pathway 37. In addition, triptolide can reverse the inhibitory effect of glioma cells on T cells and downregulate the expression of PD-L1 induced by IFN - γ 38. Therefore, triptolide can be used as an alternative molecule for glioblastoma research and drug development.

Furthermore, triptolide can also achieve anticancer effects by regulating microRNAs. Haifang Zhang et al. found that triptolide can inhibit the PI3K/AKT and Notch pathways, thereby exerting an anticancer effect on medulloblastoma cells 39. Similarly, studies have shown that triptolide can increase the expression of microRNA-181a, which participates in the proliferation, migration and apoptosis of SH-SY5Y cells, thereby exerting a tumor-suppressing effect 40.

#### 2.2.4. Pancreatic cancer

Triptolide has obvious inhibitory effects on pancreatic cancer. Triptolide, as a super-enhancer (SE) interacting agent, may exhibit its antitumor activity by interfering with cell cross-talk and signal transduction in pancreatic ductal adenocarcinoma 41.

In addition to inhibiting malignant tumors, triptolide can enhance tumor sensitivity to drugs. For example, triptolide was found to enhance the sensitivity of pancreatic cancer PANC-1 cells to GEM 42. Therefore, combined treatment modalities can offer better drug development prospects for pancreatic cancer. Studies have shown that triptolide can activate autophagy and enhance the tumor necrosis factor-related apoptosis-inducing ligand (TRAIL) sensitivity of pancreatic cancer cells 43. The drug resistance of malignant tumors is a limiting factor in the clinical application of many anticancer drugs. As a broad-spectrum anticancer drug, triptolide can inhibit the drug resistance of cancer cells, which provides a new research idea for the clinical application of triptolide and its derivatives.

In recent years, an increasing number of researchers have used nanotechnology to modify natural products to improve the efficacy of drugs and reduce side effects. For example, silk fibroin nanoparticles loaded with triptolide and celastrol have a certain synergistic effect, which includes reducing cell viability and significantly increasing the cell apoptosis rate, and may be used in a promising treatment strategy for pancreatic cancer 44. The latest research shows that triptolide can be loaded onto CRPPR peptide-modified tumor-targeting acid-triggered micelles, which can improve the therapeutic effect of triptolide and reduce damage to off-target organs 45. Therefore, it is believed that nontoxic nanomedicines based on active substances in traditional Chinese herbs have great potential as targeted and adjuvant chemotherapy for pancreatic cancer. Currently, the construction of TCM nanoformulations is providing new choices for antitumor drugs.

#### 2.2.5. Other tumors

Triptolide also has antitumor activity in other solid tumors. For example, triptolide inhibits the proliferation, invasion and migration of prostate cancer cells. When shRNA is used to silence the expression of CAV-1, triptolide can reduce the propensity of human prostate cancer cells to migrate and invade tissue 46. Triptolide inhibits the proliferation, invasion, migration and angiogenesis of oral cancer and oesophageal squamous cell carcinoma (ESCC) cells 47, 48. Triptolide can trigger the death of colon cancer cells including through apoptosis and *in vitro* experiments indicate that triptolide is effective against colon cancer stem cells (CSCs) 49. In addition, triptolide can reduce tumor-associated macrophage infiltration and inhibit the migration of colon cancer cells 50. Triptolide is a potent Nrf2 inhibitor that can inhibit the transcriptional activity of Nrf2, leading to the apoptosis of isocitrate dehydrogenase (IDH)-mutant cells, providing an operable strategy for the treatment of malignant tumors with IDH1 mutations 51. Triptolide can induce the apoptosis of cisplatin-resistant ovarian cancer cells and sensitize them to cisplatin 52. Various transcription factors, proteins and signaling pathways are involved in the antitumor effects of triptolide, but its anticancer effect is mainly achieved by inducing apoptosis.

In addition to the solid tumors mentioned above, triptolide also has a strong effect on haematological malignancies. Studies indicate that triptolide can induce cell morphological changes and exert cytotoxic effects through G0/G1 phase arrest, as well as induce apoptosis, which may be related to cross talk between components involved in apoptosis and autophagy *in vitro* 53.

Multidrug resistance (MDR) is the main obstacle to chemotherapy in the treatment of cancer, and triptolide is expected to solve this problem. Triptolide can inhibit the proliferation of A549 lung adenocarcinoma cells resistant to paclitaxel through the MAPK/PI3K/AKT signaling pathway 54. These studies indicate that triptolide has high-efficiency and broad-spectrum antitumor activity in multidrug resistant tumor cells. Triptolide also plays an important role in certain tumor cells that are resistant to radiotherapy. Triptolide can inhibit the growth and induce the apoptosis of radiotherapy-resistant nasopharyngeal carcinoma cells 55.

In addition to its roles described in the aforementioned studies, triptolide has an obvious inhibitory effect on the proliferation of pancreatic cancer, ovarian cancer, leukaemia, prostate cancer, lung cancer, liver cancer, colorectal cancer and other tumor cells, showing broad-spectrum antitumor activity. These studies have provided a theoretical basis for the pharmacological activity studies and clinical application of triptolide derivatives. At the same time, the biosynthesis of triptolide can provide a variety of precursor compounds similar to triptolide. Through interdisciplinary biosynthetic studies and pharmacological research, such as those providing precursor compounds of triptolide biosynthesis for functional research, it is possible to identify precursor compounds with anticancer effects and promote the research progress into related topics.

#### 2.2.6. The mechanism of triptolide anticancer effects

Triptolide exerts its anticancer effects by influencing apoptosis, senescence, proliferation, invasion, migration, and angiogenesis by regulating multiple signal transduction pathways and gene expression levels, as well as interactions with miRNAs and chaperones 56-59. Early studies have shown that triptolide mostly achieves anticancer effects by inducing apoptosis. Current research data show that apoptosis plays a pivotal role in the development of many tumors 60, 61. The mechanism of triptolide induced apoptosis varies by cell type. In addition to inducing apoptosis, triptolide can also affect the metabolism of tumor cells by reducing cell viability, affecting cell growth and cell cycle arrest 62, 63. Increasing evidence shows that in addition to the ability of triptolide to induce apoptosis, it can also achieve anticancer effects by inducing autophagy and the combined effects of apoptosis and autophagy. However, the relationship between apoptosis and autophagy is very complicated. Currently, there are three main reported relationships between apoptosis and autophagy: autophagy and apoptosis can cooperate to promote cell death; autophagy and apoptosis can inhibit each other; and autophagy can promote the progression of apoptosis. In addition, autophagy has a dual role in cancer cells. On the one hand, it can provide energy for cells or effective compounds to promote cell survival. On the other hand, excessive autophagy can promote the process of apoptosis 64. However, the mechanism by which triptolide induces autophagy in cancer cells and the relationship between apoptosis and autophagy have not been clearly elucidated.

In addition to apoptosis and autophagy, cell senescence, which is a form of irreversible cell growth arrest, is related to tumor treatment. Triptolide can inhibit tumor growth by inducing cell senescence 25. However, research on this effect of triptolide is still relatively limited. As a multitarget anticancer compound, triptolide can also reduce changes in mitochondrial membrane potential and lysosomal membrane permeability (LMP) 65, 66.

**Figure 4:**
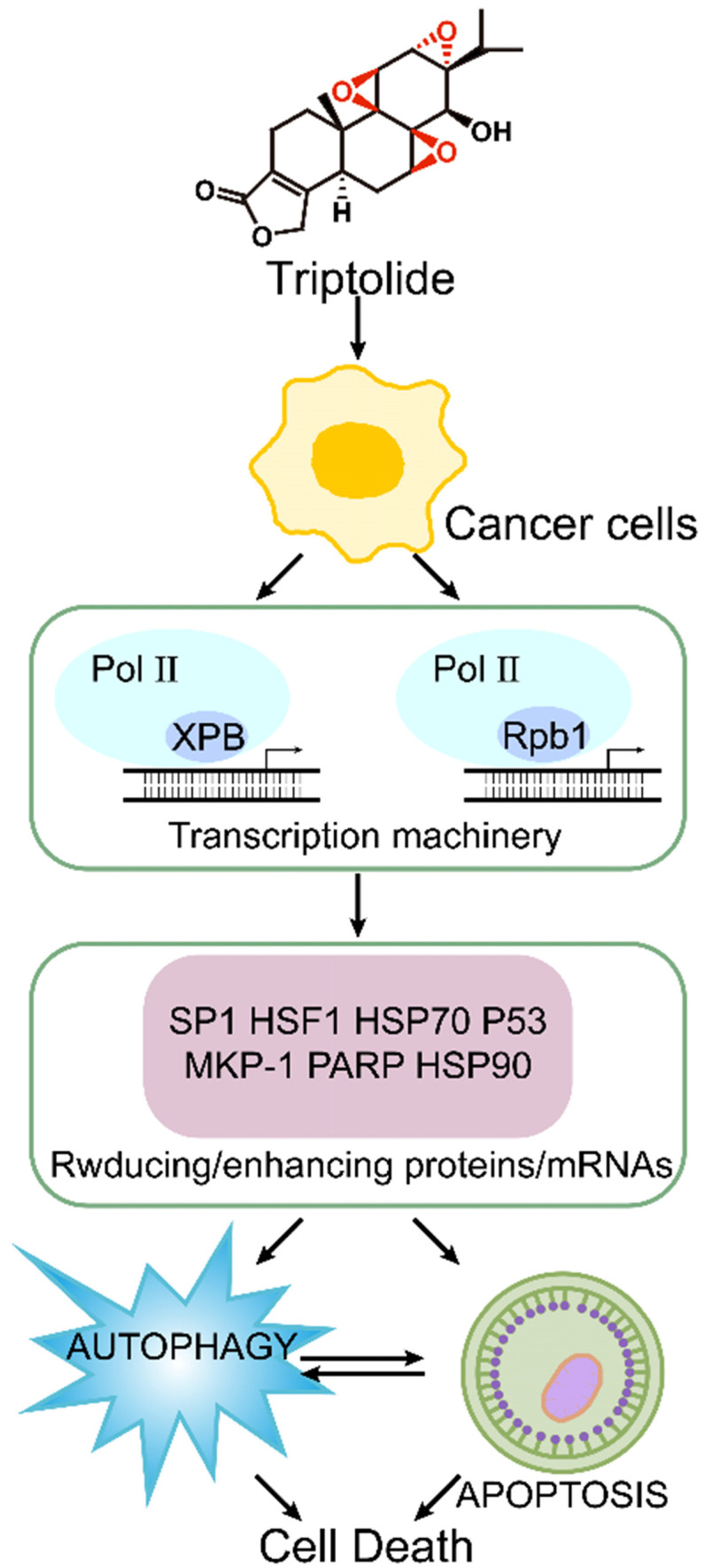
Mechanism of triptolide induced apoptosis and autophagy.

Recent research shows that the molecular target of triptolide is the XPB1 subunit of the transcription factor TFIIH 67. It primarily covalently binds to Cys342 of the XPB1 subunit via the 12,13-epoxy group, thus inhibiting RNA polymerase II-mediated transcription 68. (Figure 4) It has been reported that triptolide can degrade TFIIH in a CDK7-dependent manner, thereby causing tumor cell death 69. There are also reports that in multidrug resistant tumor cells, triptolide achieves antitumor and multidrug resistance effects through cyclin-dependent kinase 7 (CDK7), not XPB 70. Many other important transcription factors, such as Sp1 and HSF1, are also involved in the survival, development and angiogenesis of tumor cells. Cellular signal transduction pathways such as NF-κB, PI3K/Akt and CaMKK/β-AMPK are also essential for the antitumor activity of triptolide 63. In tumor cells, triptolide can effectively activate or degrade proapoptotic proteins such as heat shock proteins 90 and 70 (HSP90 and HSP70), caspase-3, caspase-9 and poly-ADP ribose polymerase (PARP) 71.

### 2.3. Other pharmacological activities

In addition to its obvious antitumor, anti-inflammatory and immunosuppressive effects, triptolide has many additional pharmacological effects that are under study. For instance, triptolide has a good effect on some neurodegenerative diseases, and it was found to improve glomerular sclerosis in patients with diabetic nephropathy.

Although the pathogenesis of the most common neurodegenerative diseases such as Alzheimer's disease (AD) and Parkinson's disease (PD) has not been clearly elucidated. Studies have confirmed that triptolide has certain neuroprotective and neurotrophic effects in AD 72. In addition, triptolide can upregulate mGlu5 to inhibit the activation of microglial cells and induce reactive astrocytes, which in turn protect dopaminergic neurons in a PD model 73.

Triptolide can inhibit the binding of p53 to the promoter of GADD45B to downregulate its transcription. Inhibiting p53-NF-κB-GADD45B signaling to maintain glomerular barrier function provides new research ideas for the anti-proteinuria effect of triptolide in glomerular diseases 74, 75. In addition, triptolide may improve the proteinuria of diabetic rats by inhibiting the PDK1/Akt/mTOR pathway 76. The latest research shows that triptolide can inhibit the PI3K/AKT signaling pathway and the interaction between miR-188-5p and PTEN to treat diabetic nephropathy 77.

## 3. Biosynthesis of triptolide

Following the rapid development of new tools in recent years, synthetic biology has been successfully applied to the production of artemisinin, paclitaxel (Taxol^®^) and other active compounds isolated from TCM materials. The use of synthetic biology principles to design and modify microbial strains to produce natural active substances has become a very promising method for obtaining sufficient quantities of natural products. This approach is also expected to enable the efficient industrial production of triptolide precursors, triptolide and its derivatives in the future.

### 3.1. Genomic and transcriptomic studies of *T. wilfordii*

To explore the key genes of triptolide biosynthesis, our team analyzed tissue samples of *T. wilfordii* leaves, flowers, stem bark, peeled stem, root bark, root phloem and root xylem, as well as suspensions of cells treated with methyl jasmonate (MeJA) for different times. The samples were classified according to the principle of three biological repeats. The total RNA of each sample was extracted by a modified cetyltrimethylammonium bromide (CTAB) method for sequencing analysis, and finally the transcriptome data were obtained 78. The results showed that roots and leaves had the highest triptolide content 79. Therefore, the key genes of triptolide biosynthesis can be screened according to the correlation of their differential expression in different tissues with the triptolide content. Moreover, induction with MeJA increased the content of triptolide in suspension cells. By analysing the expression of genes in suspension cells induced by MeJA at different times, the key genes that regulate triptolide biosynthesis were identified.

In addition, our research team sequenced the genome of *T. wilfordii*. A total of 28321 protein coding genes were annotated, with an average sequence length of 3338 bp. On average, each predicted gene contained 5.44 exons, with a total of 182.52 Mb of annotated repeats, accounting for 52.36% of the *T. wilfordii* genome 78. Based on the genomic data, evolutionary events in the life history of *T. wilfordii* were analyzed. It was found that the most recent WGT events included the duplication of genes in the upstream metabolism of isoprene. These results suggested that recent WGT events are of great significance to the evolution of triptolide biosynthesis.

The genome and transcriptome, as the main tools for screening biosynthetic pathway genes, have some limitations. In the genome, when identifying genes of the same family, it is possible to merge the genes with high similarity into one gene, which is likely to lead to mistakes in the screening process. In the process of cloning target genes, the gene sequence provided by the genome is mainly the open reading frame (ORF) of the gene. Therefore, if the expression level of the gene is low, the target gene may not be identified due to the limitations of the primers. In addition, the gene sequences provided by the transcriptome may have splicing errors or gene sequence deletion problems. Therefore, it is necessary to integrate the gene information provided by the transcriptome and genome for better screening and cloning of target genes.

### 3.2. Analysis of the upstream terpenoid biosynthesis pathway

The carbon backbone of terpenes is mainly formed by condensation of isoprene pyrophosphate (IPP) and dimethylallyl diphosphate (DMAPP). In nature, IPP and DMAPP are synthesized in two different biochemical pathways, the 2*C*-methyl-D-erythritol-4-phosphate (MEP) pathway and the mevalonate (MVA) pathway (Figure 4). Moreover, the MEP and MVA pathways provide precursors for different terpenoids. The MEP pathway mainly produces mono-, di- and tetraterpenes, while the MVA pathway mainly produces sesquiterpenes, sterols, triterpenes and their saponin derivatives.

#### 3.2.1. The mevalonic acid (MVA) pathway

The MVA pathway was first discovered by Lynen et al. 80 and elaborated by Newman et al. 81. This pathway mainly produces IPP through a series of 6 enzymatic reactions, as follows: ⅰ) The pathway is initiated with acetyl-CoA thiolase (ACAT) catalyzing the formation of acetoacetyl-CoA from two acetyl-CoA units 82. ⅱ) HMG-CoA is generated from acetoacetyl-CoA and a third acetyl-CoA unit catalyzed by 3-hydroxy-3-methylglutaryl-CoA synthase (HMGS) 83. ⅲ) The main regulatory step of the MVA pathway is the reduction of HMG-CoA to mevalonate by HMG-CoA reductase (HMGR) on the surface of the endoplasmic reticulum (ER) 84. ⅳ) Mevalonate is phosphorylated by mevalonate kinase to mevalonate-5-phosphate 84. ⅴ) Subsequently, phosphomevalonate kinase phosphorylates mevalonate-5-phosphate to mevalonate-5-diphosphate 85. ⅵ) The final step of the MVA pathway is the formation of IPP from mevalonate diphosphate by mevalonate diphosphate decarboxylase 86. IPP can be reversibly isomerized to DMAPP by IPP isomerase (IPI).

Two AACT genes, one HMG gene, two HMGR genes, one MVK gene, one PMK gene and one MVD gene were cloned from *T. wilfordii*. The predicted number of amino acid residues, the ORF sequence, theoretical isoelectric point (pI), molecular weight and the number of genes were analyzed 87. In addition, some scholars confirmed the gene function by introducing cloned *TwHMGS* into a suitable yeast strain, and then studying the inducible expression and tissue expression patterns 88.

#### 3.2.2. The 2-C-methyl-d-erythritol-4-phosphate (MEP) pathway

In 1999, Rohmer et al. discovered another route for the synthesis of terpenoids, the methylerythritol phosphate (MEP) pathway. This pathway was elucidated by Lichtenthaler et al. 89 and is also called the 1-deoxy-D-xylulose-5-phosphate (DXP) pathway. Under the catalysis of 7 enzymes, D-3-phosphoglyceraldehyde (GAP) and pyruvate are condensed and reduced to produce IPP and DMAPP via the following steps: ⅰ) Pyruvate and GAP are condensed to form 1-deoxy-d-xylulose 5-phosphate (DXP) under the catalytic action of DXP synthase (DXS). DXS is a key enzyme that controls the flux of the MEP pathway 90. ⅱ) DXP reductoisomerase (DXR) catalyzes the isomerization of DXP to form MEP 91. ⅲ) 2-C-methyl-d-erythritol 4-phosphate cytidyltransferase (MCT) activates MEP to form CDP-ME with a cytidine diphosphate linkage at the C4 position 92. ⅳ) CDP-ME is phosphorylated into CDP-MEP under the action of 4-diphosphocytidyl-2-C-methyl-D-erythritol kinase (CMK). ⅴ) Subsequently, MDS cyclizes the activated CDP-MEP to 2-C-methyl-d-erythritol-2,4-cyclodiphosphate (MEcDP) 93. ⅵ) MEcDP is reduced to 1-hydroxy-2-methyl-2-(E)-butenyl-4-diphosphate (HMBDP) by 4-hydroxy-3-methylbut-2-enyl diphosphate synthase (HDS) 94. ⅶ) In the final step, HMBDP is converted by 4-hydroxy-3-methylbut-2-enyl diphosphate reductase (HDR) to form IPP and DMAPP at a product ratio of approximately 5:1 95. In addition, IPI is also present in plastids, and can catalyze the isomerization of IPP to maintain the optimal ratio of IPP to DMAPP 96.

Two *TwDXS* and one* TwDXR* gene were cloned from *T. wilfordii*, and the researchers verified their functions through colour complementation experiments 97. Further overexpression (OE) and RNA-interference experiments confirmed that the expression of *TwDXR* affects the production of triptolide in *T. wilfordii* 98. The *TwHDR* gene encodes the final enzyme of the MEP pathway, which is very important for regulating isoprene biosynthesis. The function of* TwHDR* was verified by a complementation assay in a mutant strain of *E. coli* with a defective HDR gene, and the expression of *TwHDR* in the cells in suspension was analyzed, and the results contributed to further analysis of the triptolide biosynthesis pathway 99.

### 3.3. Analysis of the triptolide biosynthesis pathway

**Figure 5:**
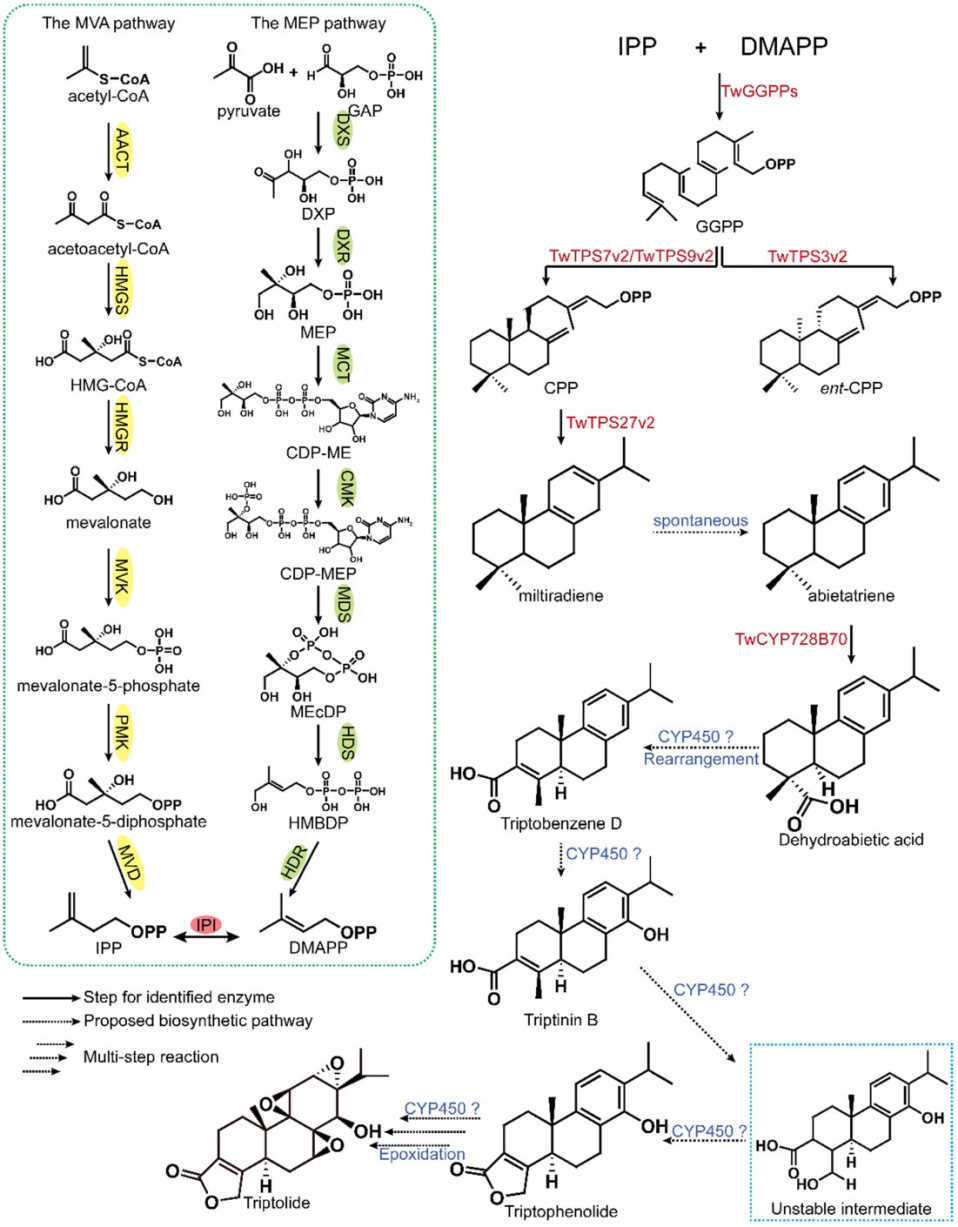
Analysis of the biosynthetic pathway of triptolide. The green dashed box shows the common upstream pathways of terpenoids in *T. wilfordii*. The solid arrow and red gene indicate the route of identified function, while the dotted arrow and blue gene indicate the possible route.

The biosynthesis of triptolide is mainly divided into three steps. The first step encompasses the production of IPP and DMAPP through the MVA and MBP pathways 100, 101. The second step consists of the formation of geranylgeranyl diphosphate (GGPP) by geranylgeranyl diphosphate synthase (GGPPS), catalyzing the continuous addition of IPP to DMAPP, geranyl pyrophosphate (GPP) and farnesyl pyrophosphate (FPP) 102. In the final step, GGPP is converted to various terpene intermediates under the catalysis of diterpene synthases (diTPSs), and the intermediates are then modified by different postmodification enzymes to generate the corresponding diterpene products (Figure 5) 103.

GGPPS can catalyze the generation of the common diterpene precursor GGPP and is considered to be one of the key synthetases in the diterpene biosynthesis pathway. Five putative *GGPPS* genes have been cloned from *T. wilfordii*. The cloned *GGPPS* and *SmCPS/KSL* genes were introduced into E. coli with miltiradiene serving as a marker. Finally, it was determined identified that the proteins encoded by the three *TwGGPPS* exhibited function of GGPPs. By analysing the expression in *TwGGPPS* in MeJA-induced cells in suspension, researchers showed that the accumulation of triptolide is enhanced with the increase of *TwGGPPS1* and *TwGGPPS4* expression, suggesting that these two genes may be the main genes that control triptolide synthesis 104. The latest research shows that *TwGGPPS8* exhibits an expression pattern similar to that of *TPS7v2*, *TPS9v2* and* TPS27v2*, and the highest transcription levels were found in roots rich in triptolide. Based on this observation, it was speculated that *TwGGPPS8* may be involved in the biosynthesis of triptolide in roots 105.

After obtaining the common linear diterpene precursor GGPP, researchers further studied the biosynthetic pathway of triptolide. Hansen et al. found that *TwTPS27* coupled with *TwTPS9* converted normal copalyl diphosphate to miltiradiene by screening diterpene synthase family genes in *T. wilfordii* 106. Su et al. added miltiradiene to the culture medium of suspended cells, and the accumulation of triptolide after 5 days exhibited a statistically significant increase compared with the level in the control group 79. This is the first evidence that miltiradiene is indeed a precursor of triptolide. Through transcriptome sequencing of cells in suspension induced with MeJA, 8 putative diterpene synthase genes were identified, and 6 full-length diterpene synthase genes were cloned. Using GGPP as a substrate, the functional identification was carried out in *E. coli*, and *TwTPS7v2* and *TwTPS9v2* were confirmed to generate CPP from GGPP 79. It was confirmed that CPP is a precursor of miltiradiene. The results showed that TwTPS27v2 can catalyze the formation of miltiradiene from CPP, and RNAi experiments showed that decreased expression of *TwTPS7v2*, *TwTPS9v2* and *TwTPS27v2* affects the accumulation of triptolide.

Previous studies had elucidated the biosynthesis of the abietane-type diterpene core skeleton miltiradiene, which laid the foundation for further investigation of cytochrome P450 (CYP450) genes in the downstream synthesis pathway. NADPH-cytochrome P450 reductase acts as the electron donor of CYP450 proteins to support their catalytic function. Four *TwCPR* genes were cloned from *T. wilfordii* and soluble proteins were successfully expressed. The activity of TwCPR enzymes was verified by combining them with kaurene oxidase. The results showed that although *TwCPR3* was expressed at lower levels in certain tissues, it was a more efficient electron donor 107. Therefore, it was speculated that TwCPR3 is more suitable for the study of other CYP450 monooxygenases in *T. wilfordii*. Studies have reported that CYP720B4 in Sitka spruce (*Picea sitchensis*) can convert miltiradiene to dehydroabietic acid, and it was speculated that dehydroabietic acid may be an important intermediate in the triptolide biosynthesis pathway 108. The latest research indicates that CYP728B70 is the first CYP450 in the triptolide biosynthesis pathway and that it converts miltiradiene and abietatriene in two consecutive oxidation steps to form the corresponding diterpene alcohol and diterpene acid (dehydroabietic acid) products. Interference and OE analysis indicated that CYP728B70 is involved in triptolide biosynthesis 78.

Currently, there has been a breakthrough in the understanding of the triptolide biosynthesis pathway, and the first CYP450, *TwCYP728B70*, was discovered. However, there are still many difficulties to be resolved. First, compared with triptolide, the position of the carboxyl group of dehydroabietic acid is problematic. Transfer of the carboxyl group to the three position is an urgent problem for researchers. On the one hand, after decarboxylation, a methyl group may be attached to the third position, and then the three-step oxidation proceeds. On the other hand, there may be an enzyme that can directly transfer the carboxyl group at position 18 to position 3. In addition, the mechanism involved in forming the three epoxy groups in triptolide has not been extensively studied. As suggested in the current literature, CYP450s and dioxygenase may catalyze the formation of these functional groups. Therefore, we hope to solve the problems of carboxyl transfer and epoxy group formation during biosynthesis by combining biosynthesis with chemical synthesis, and ultimately enable the industrial production of triptolide.

### 3.4. Metabolic engineering for achieving triptolide biosynthesis

Studies have shown that the content of triptolide in *T. wilfordii* is low, reaching only 66.5 μg/g 5. To study triptolide better, researchers used *Agrobacterium rhizogenes* to induce hairy roots from the calli of *T. wilfordii* roots. The concentrations of triptolide in hairy root, callus, adventitious root and natural root samples were 39.98, 1.76, 47.86 and 21.4 μg/g, respectively, and after MeJA induction, the triptolide concentration increased approximately 2-fold 109. In addition, by optimizing the growth and induction conditions for* T. wilfordii* adventitious root culture, a comparatively large quantity of triptolide was obtained. The yield of triptolide was increased to 648.3 μg per flask by adding inducers XAD-7 and MeJA and optimizing the growth conditions 110.

*T. wilfordii* cells in suspension are also important sources of triptolide for research. Suspension cells are also suitable for a variety of experiments, such as RNAi and overexpression studies. In one study, the triptolide concentrations in *T. wilfordii* cells in suspension and culture medium were 53±3 μg/g and 4.0±0.2 mg/L, respectively. After 240 h of MeJA induction, the triptolide content was increased 3-fold to 200 μg/g 79, 111. Furthermore, researchers found that cambial meristematic cells (CMCs) also effectively accumulate triptolide, which has great potential in triptolide biosynthesis research. Studies have shown that CMCs contain triptolide at a concentration of 138.1 μg/g, and after induction with MeJA for 480 h, the content of triptolide can reach 405.1 μg/g 112. Subsequently, researchers confirmed that MeJA can induce the expression of terpene biosynthesis-related genes in CMCs through real-time reverse transcription-polymerase chain reaction (qRT-PCR). CMCs can therefore be used for the analysis of the triptolide biosynthesis pathway.

### 3.5. Metabolic engineering of bacteria for triptolide production

Microbial metabolic engineering is a very promising method for obtaining natural products. Miltiradiene is an important intermediate compound of triptolide biosynthesis. The synthesis of miltiradiene by microorganisms is the first step to efficiently produce triptolide. Studies have shown that modular engineering, encompassing the integration of *Sm*CPS and *Sm*KSL together with the integration of BTS1 and ERG20, significantly contributed to the increased output of miltiradiene. Finally, the best synthetic route was introduced into the diploid yeast strain YJ2X, and the resulting engineered strain produced 365 mg/L miltiradiene in a 15-L bioreactor 113. In addition, Dai et al. increased the yield of miltiradiene to 488 mg/L through various methods, such as overexpression of key enzymes and the use of antibiotic markers to replace auxotrophic markers in plasmids. However, due to the use of antibiotics in the fermentation process to enhance the stability of the plasmid, it cannot be used in large-scale industrial production 114. Recently, Tianyuan Hu et al. investigated the production capacity of diterpenoid synthases from different species, and selected a class II diterpene synthase (di-TPS) *Cf*TPS1 from *Coleus forskohlii* (*Plectranthus barbatus*) and class I di-TPS *Sm*KSL1 from *Salvia miltiorrhiza* to use for producing a final titre of miltiradiene of 3.5 g/L in a 5-L bioreactor 115.

In addition, researchers knocked out *rox1*, *ypl062w*, and *yjl06w4*, downregulated *ERG9*, and overexpressed genes such as *tHMG1* and *ERG20* to increase GGPP production. Subsequently, they introduced double *SmMS-SmCPS1* fusion modules to produce miltiradiene and finally co-expressed the *TwCYP728B70* and *TwCPR3* genes to produce dehydroabietic acid 78. This series of experiments laid the foundation for the subsequent identification of key enzyme-coding genes in the triptolide biosynthesis pathway.

Although a microbial metabolic plant model has been constructed to produce dehydroabietic acid, it is difficult to meet the needs of subsequent research because of its low yield. Currently, there are several ways to improve the yield of synthetic biology: 1. Genes that do not affect the growth of microorganisms are knocked out or weakened in other ways to increase the accumulation of precursor compounds. 2. The yield of target compounds is increased by the overexpression of genes. 3. Genes with the same function but with higher activity are used to replace genes with lower expression or mutation technology is used to identify mutant genes that produce higher yields. 4. Through the technology of protein fusion or substrate channelization, we can connect the active pockets of proteins to improve the yield of target compounds.

## 4. Total synthesis of triptolide

Over decades, relatively slow progress has been made toward the goal of total triptolide synthesis, with many of the advancements made attributed the efforts of Berchtold 116, van Tamelen 117 and Yang 118. Currently, there are four principal ways to synthesize triptolide: Ⅰ) synthesis from tetralinone, α-abietic acid or α-dehydroabietic acid as starting materials, Ⅱ) synthesis via the Diels-Alder reaction, Ⅲ) synthesis of the core skeleton via a polyene cyclization reaction, and IV) synthesis via metal catalysis 119.

**Scheme 1:**
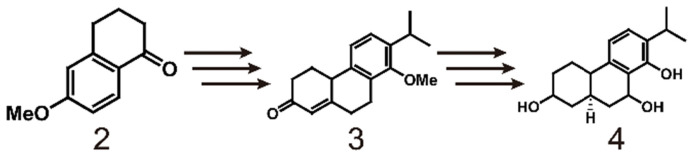
Early research on the synthesis of triptolide using 6-methoxy-1-tetraketone (2) as starting material.

**Scheme 2:**
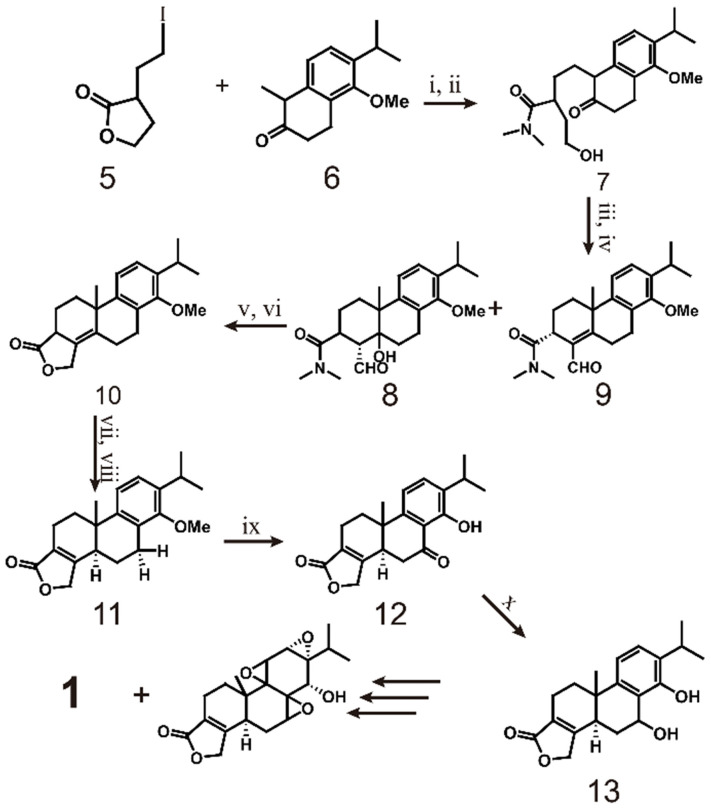
Synthesis of triptolide (**1**) through **5** and **6**. ⅰ) NaH, DMF 25℃, 12h; ⅱ) Me_2_NH 25 ℃, 12 h; ⅲ) CrO_3_.py, CH_2_Cl_2_ 25 ℃, 15 min; ⅳ) grade 3 neutral alumina EtOAc, 25 ℃, 48 h; ⅴ) pˑTosOH (catalyst) C_6_H_6_, reflux, 2 h; ⅵ) NaBH_4_, EtOH, 25 ℃, 2 h aqueous HCl (workup); ⅶ) MeO^-^, MeOH 25 ℃, 15 min; ⅷ) CrO_3_, HOAc-H_2_O (9:1) 25 ℃,6 h; ⅸ) BBr_3_, CH_2_Cl_2_ 25 ℃,10 h; ⅹ) NaBH_4_, EtOH 25 ℃,1 h.

As early as 1977, the Berchtold team began research on the total synthesis of triptolide (scheme 1). They used 6-methoxy-1-tetralone (**2**) as the starting material and converted it to tricyclo-enone **3** in 10 steps with a yield of 33%. Then, **3** was transformed into **4** in 5 steps, which laid the foundation for the total synthesis of triptolide 120. In 1980, the team completely outlined the process of synthesizing racemic triptolide (scheme 2) 116. The reaction is initiated with the alkylation of tetralone (**5**) with 3-(2-iodoethyl) dihydrofuran-2(3H)-one (**6**), and bonds in the product are broken with dimethylamine to form a 1:1 enantiomer mixture **7**. The mixture of **8** and **9** is formed by oxidation of **7** with a CrO_3_.py complex with aldol condensation catalyzed by Al_2_O_3_. To obtain pure compound **10**, the mixture is treated with acid to dehydrate **8**, the aldehyde is reduced with NaBH_4_ during the acid treatment, and an acid-catalyzed lactonization reaction is performed. The isomerization of **10** catalyzed by methoxide results in compound **11**, which is then oxidized by benzoic acid **11** and demethylated to obtain **12**. Reduction of **12** with NaBH_4_ provides pure C-7β alcohol (**13**). Finally, triptolide (**1**, 21%) and 14-epitriptolide (68%) are obtained through an Alder periodate reaction (NaIO_4_, 74%), a sequencing m-CPBA oxygenation and basic hydrogen peroxide oxygenation (H_2_O_2_/OH^-^) procedure, and sodium borohydride reduction 119. In summary, the first total synthesis of racemic triptolide was completed from **5** in 16 steps. Although the final yield was low (1.6%), this work undoubtedly laid the foundation for the study of triptolide synthesis.

**Scheme 3:**
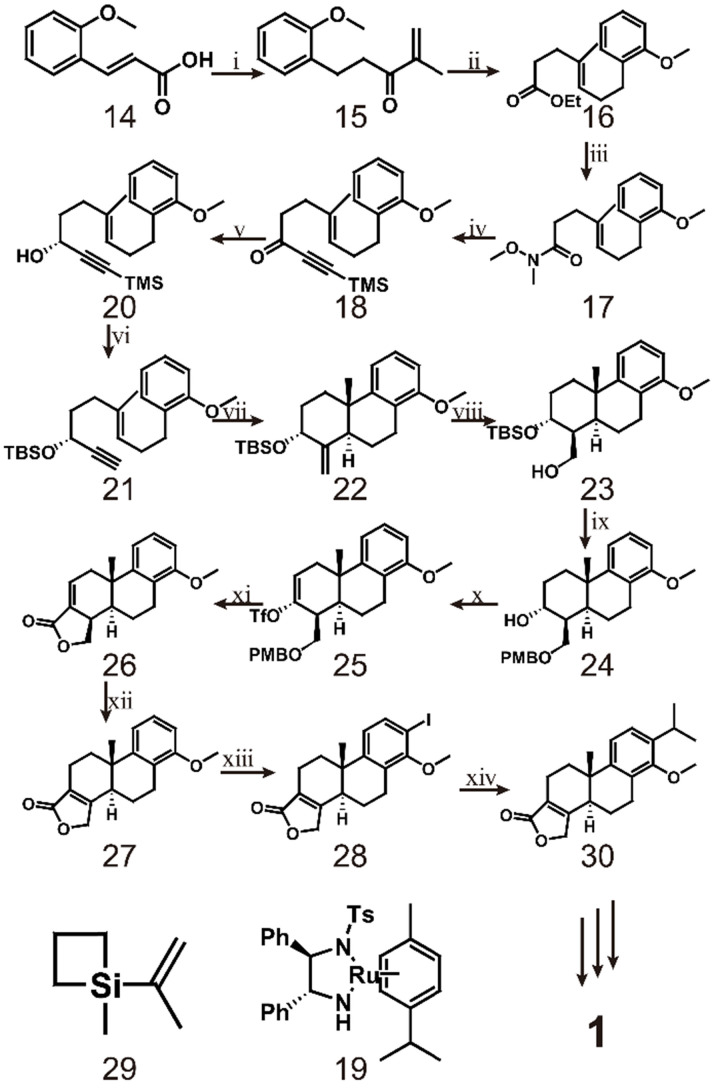
Synthesis of triptolide (**1**) through Compound **14**. ⅰ) H_2_, Pd/C, NH(OMe)MeˑHCl, CDI, Isopropenyl magnesium bromide; ⅱ) NaBH_4_, CeCl_3_ˑ7H_2_O, CH_3_C(OEt)_3_, CH_3_CH_2_COOH, reflux; ⅲ) LiOH, NH(OMe)MeˑHCl, CDI; ⅳ) Trimethylsilyl lithium acetylide, -78 ℃ to -20 ℃; ⅴ) compound 19, HCOOH, TEA, THF; ⅵ) K_2_CO_3_, TBSCI, imidazole; ⅶ) InBr_3_ (20%), -20 ℃; ⅷ) BH_3_ˑTHF, H_2_O_2_, 3N NaOH; ⅸ) NaH, PMBCI, PTSA; ⅹ) Jones reagent, LiHMDS, Tf_2_NPh, -78 ℃ to rt; ⅹⅰ) DDQ, CO, Pd(PPh_3_)_4_, Bu_3_N, LiCl, 80 ℃; ⅹⅱ) Rh(PPh_3_)_3_Cl, Et_3_SiH, toluene reflux; ⅹⅲ) I_2_, AgOTf; ⅹⅳ) Pb(dba)_2_, TBAF, compound 29, 5% Pd/C.

Later, researchers mostly borrowed from the research ideas of Berchtold et al. The innovation of the synthetic route was mainly focused on different treatment methods of tetralone. However, Li et al. developed a different route to synthesize triptolide in 2014 121 (scheme 3). The route starts from the hydrogenation of common compound **14**, which is converted to the corresponding Weinreb amide and finally reacts with isopropenyl magnesium bromide to form enol **15**. Compound **15** is then reacted with sodium borohydride in the presence of CeCl_3_, after which Johnson Claisen rearrangement produces ester **16**. This ester is hydrolysed to form amide **17**, which is then reacted with trimethylsilyl lithium acetylide to obtain acetylene **18**. Compound **18** and 2.5 mol% of (R, R)-**19** are incubated in triethylamine and formic acid for 1.5 h to produce alcohol **20**. Compound **21** is obtained by protecting the hydroxyl group with a *tert*-butyldimethylsilyl ether during the potassium carbonate/methanol repair process and then cleaving the acetylenic trimethylsilyl group. The key to this synthetic pathway is that indium-(III) catalyzes the cationic cascade reaction of compound **21**. This reaction proceeds via slow addition of **21** to an intensely stirred suspension of InBr_3_ in dichloromethane at -20 °C. Under these conditions, key intermediate **22** is formed as a single isomer. Subsequently, the authors completed the synthesis of the lactone D-ring through a four-step reaction. In the first step, **22** was subjected to hydroboration using a BH_3_·THF complex and then oxidized with basic hydrogen peroxide to obtain alcohol **23** as a single isomer. In the second step, PMB ether was formed to protect the free hydroxyl group of alcohol **23**, and then *p*-TsOH was used to remove the silyl protecting group to obtain alcohol **24**. The third step is to oxidize **24** with Jones reagent and then convert the resulting carbonyl group to the corresponding trifluorovinyl compound **25**. Finally, the PMB ether of** 25** was oxidatively cleaved, and tetracyclic lactone **26** was obtained by palladium-catalyzed carbonylation and *in situ* lactonization. After obtaining intermediate **26**, compound **27** was obtained by reacting 26 with a catalytic concentration of tris(triphenylphosphine)rhodium and triethylsilane in refluxing toluene. Ortho-iodination of **27** gave intermediate **28**, which was subjected to palladium-catalyzed cross-coupling with 1-methyl-1-(prop-2-enyl) silacyclobutane **29** and then hydrogenated to form triptophenolide methyl ether **30**. Triptophenolide methyl ether, as an important intermediate, has been used in the total synthesis of triptolide many times 118, 122. The difficulty of this approach is the preparation of optically active propargyl alcohol (R)-**20**. Through several attempts by researchers, the conditions for incubation of **18** and 2.5 mol% of (R,R)-**19** in triethylamine and formic acid for 1.5 h were finally discovered 123. It is speculated that these conditions may a result of the instability of the trimethylsilyl group in the reaction system. Research mainly utilize InBr_3_-mediated cationic polyene cyclization and palladium-catalyzed carbonylation with lactone formation, which enables the large-scale synthesis of intermediate **26** 121.

**Scheme 4:**
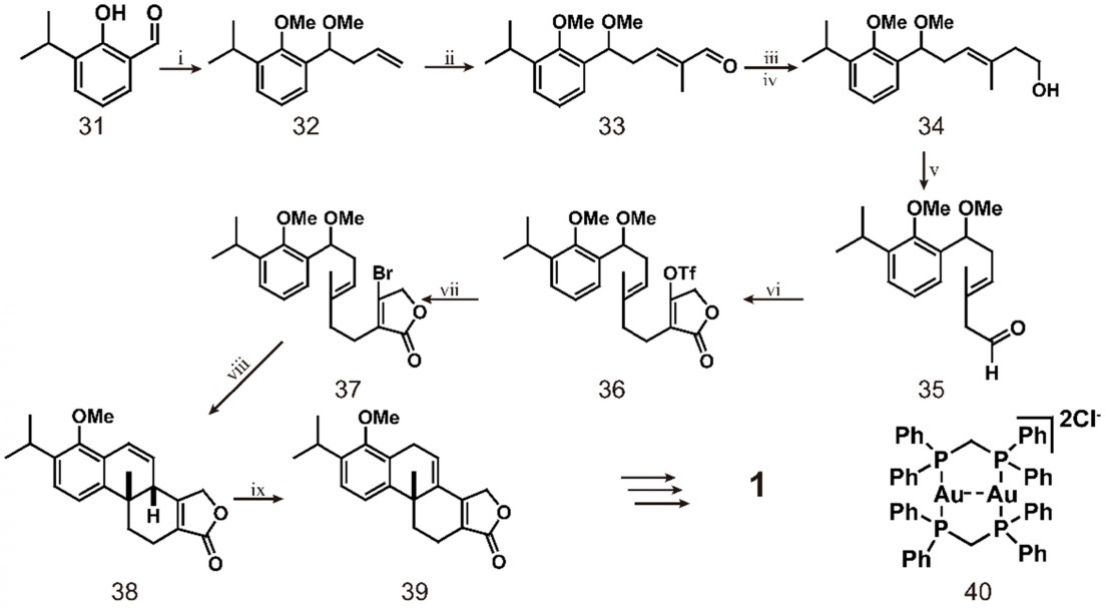
Total synthesis of triptolide (**1**) through simple compound **31**. ⅰ) allylMgBr, THF, Me_2_SO_4_, 70 ℃ ,12 h; ⅱ) Grubbs Ⅱ (1 mol%), Cul (2.5 mol%), Acrolein, Et_2_O, 40 ℃, 4 h; ⅲ) Ph_3_PMel, KHMDS, THF, 2 h; ⅳ) Cy_2_BH, THF, NaBO_3_ˑ4H_2_O, 4 h; ⅴ) DMP, CH_2_Cl_2_; ⅵ) L-proline (5 mol%), tetronic acid, Hantzch ester CH_2_Cl_2_, DIPEA, Tf_2_O, -78 ℃; ⅶ) LiBr, THF, 70 ℃, 12 h; ⅷ) compound 40, Na_2_CO_3_, 365 nm, MeCN, 12 h, H_2_SO_4_, 6 h; ⅸ) RuCl_2_(PPh_3_)_3_, DIPEA, PhMe, 120 ℃, 4 d.

Recent studies have used dimeric gold complex [Au_2_(dppm)_2_] Cl_2_ combined with ultraviolet A (UVA, 365 nm) light irradiation to catalyze the formation of radical intermediates from non-activated bromoalkanes/arenes in a mild photoredox catalysis process 124 (scheme 4). The first step of this reaction is treating aldehyde **31** with allyl magnesium bromide and then adding dimethyl sulfate to obtain allyl **32**. Compound **32** is subjected to a metathesis reaction with methacrolein under the catalytic action of a Grubbs second-generation catalyst and a certain amount of copper iodide to obtain a single isomer of aldehyde **33**. The aldehyde groups of compound **33** are converted to a diene by the Wittig reaction and then to the corresponding primary alcohol **34** by a borohydride/oxidation sequence. Dess Martin oxidation of **34** produces aldehyde **35**, which is subjected to a one-pot proline-catalyzed coupling reaction with tetronic acid in the presence of Hantzsch ester and triflation to afford triflate **36**. The mixture is directly heated with lithium bromide in tetrahydrofuran to obtain bromobutene lactone **37**, which reacts under optimal conditions, followed by treatment with sulfuric acid to obtain tetracycle** 38**, and the configuration is confirmed by X-ray analysis. Subsequently, **38** is isomerized using a catalytic amount of RuCl_2_(PPh_3_)_3_ and DIPEA in toluene at 120 °C to obtain **39**. Finally, the conversion of **39** to triptolide requires 8 more steps 125. This route can be used to access the important intermediate **39** for triptolide synthesis, which is converted from compound **31** in eight simple steps. However, the difficulty of the entire route is maintaining the optimal conditions for the reaction of bromo-butyrolactone **37**. The researchers finally determined that, vinyl bromide (0.1 M in MeCN), 1 (10 mol%), Na_2_CO_3_ (2 equiv.), subjected to, 365-nm LED light for 12 h at room temperature constituted the best reaction conditions. This study has made important contributions to the large-scale synthesis of polycyclic compounds and can be used in the synthesis of many other terpenoids.

The total synthesis of triptolide mainly includes the following three aspects: i) the synthesis of the tricyclic scaffold; ii) the formation of the butenolide (D-ring), and iii) the construction of the three active epoxy groups. Previous research on the total synthesis of triptolide has solved these three problems in a satisfactory manner and achieved important research results on a laboratory scale. However, in view of the complex chemical structure of triptolide, even as researchers continue to optimize the synthetic pathway and reduce the number of steps required for its total synthesis, the final yield of triptolide remains too low. Therefore, researchers need to make unremitting efforts to develop new approaches for triptolide synthesis.

## 5. Toxicity of triptolide

With the increasing clinical application of* T. wilfordii* and triptolide, increasing numbers of studies and clinical case reports indicate that triptolide has serious adverse effects. Currently, triptolide has a narrow therapeutic window and induces serious toxicity and side effects, which limits its clinical application. Considering this information, we have summarized the research progress on the hepatotoxicity, nephrotoxicity, cardiotoxicity and reproductive toxicity of triptolide, hoping to contribute to better clinical prospects of this compound.

### 5.1. Hepatotoxicity

Liver injury is the most common adverse reaction caused by triptolide, and has caused widespread concern. Many studies have been carried out to explain the mechanism of triptolide-induced liver toxicity, mainly focusing on common phenomena such as oxidative stress and inflammation 126, 127. In recent years, researchers have discovered that mitotic phagocytosis associated with mitochondrial fission may be a new mechanism of induced triptolide hepatotoxicity 128. Jie Zhao et al. analyzed triptolide-induced changes in the serum and liver metabolome in mice, identified 30 metabolites that were significantly changed, and selected 29 of these metabolites as potential biomarkers related to triptolide-induced hepatotoxicity, with the aim of helping researchers better understand the mechanism of triptolide-induced toxicity 129. In addition, proteomics and targeted fatty acid analyzes were also used to reveal the mechanism of triptolide hepatotoxicity.

Yan Lu et al. found that triptolide can reduce the transcription of CYP3A, CYP2C9, CYP2C19 and CYP2E1, and the substrate affinity of the proteins leads to liver toxicity 130. CYP3A is the main isozyme involved in triptolide metabolism; it facilitates the detoxification of triptolide. Experiments show that catalpol (CAT), the main component of *Rehmannia glutinosa*, can increase the expression of detoxification enzymes CYP3A2/4, CYP2C9 and UGT1A6, increase the metabolic transformation of triptolide, and thereby reduce its liver toxicity 131. Knocking out hepatic cytochrome P450 caused a significant increase in triptolide levels, which aggravated its hepatotoxic effects.

In recent years, researchers have used high-content analysis (HCA) to measure the overall cytotoxicity phenotype of HepG2 cells treated with triptolide and finally confirmed that inhibition of global transcription associated with RNA Ⅱ is the core trigger of hepatotoxicity induced by triptolide 132. The latest research found that propionate produced by the intestinal flora can promote the protective effect of intestinal flora against triptolide by reducing inflammation levels 133.

### 5.2. Nephrotoxicity

The nephrotoxicity of triptolide also limits its clinical application. However, the mechanism of this toxicity has not been fully elucidated. Researchers used collagen-induced arthritis (CIA) model rats as the research objects and found that triptolide transport is mediated by OTC2 in rat kidney slices and HEK-293T cells. TNF-α can increase the toxicity of triptolide and regulate the expression and function of OTC2, thus indicating that OCT2 mediates the nephrotoxicity of triptolide *in vitro* 134.

Wei Huang et al. combined network pharmacology and targeted metabolomics, and identified 61 action targets related to renal toxicity induced by triptolide, including 39 direct targets and 22 indirect targets, and finally confirmed dihydroorotate, thymidine, 2-deoxyinosine, uric acid, adenosine and xanthine as biological markers of renal toxicity 135. In addition, the purine metabolism pathway, toll like receptor signaling pathway and NF-κB signaling pathway play key roles in the nephrotoxicity induced by triptolide.

### 5.3. Cardiotoxicity

Research by Shurong Wang et al. showed that triptolide caused an increase in the expression of more than 108 microRNAs in the heart of male rats by more than twofold and reduced AhR levels in the myocardium and circulation, inducing acute cardiotoxicity 136. Therefore, circulating AhR levels and microRNA levels can be used as early warning biomarkers for triptolide-induced cardiotoxicity.

Researchers have studied the role of p53 in triptolide-induced cardiotoxicity in H9c2 cells, primary cardiomyocytes, and C57BL/6-derived p53 mouse models 137. The results showed that Bax, a target protein of p53, leads to important mitochondrial dysfunction and apoptosis in triptolide-induced cardiotoxicity and can block the permeability of the mitochondrial membrane to protect against triptolide-induced myocardial toxicity.

### 5.4. Reproductive toxicity

Triptolide has strong reproductive toxicity, mainly in males. Triptolide can inhibit spermatogenesis and testosterone marker enzymes, reduce sperm count, lower the gonadal index and destroy the testicular microstructure 138. Bo Ma et al. evaluated the mechanism of triptolide-induced reproductive toxicity and identified possible new biomarkers 138. They reported that triptolide-mediated downregulation of PPAR caused abnormal testicular lipid and energy metabolism, which led to sperm damage, revealing the mechanism of the reproductive toxicity induced by triptolide.

Recently, researchers analyzed the expression profiles of lncRNAs/circRNAs/mRNAs and revealed the mechanism of the reproductive toxicity induced by triptolide relating to lncRNAs/circRNAs 139. The results show that triptolide can reduce sperm production, lead to abnormal testicular and sperm morphology, and induce mature sperm dysfunction. After stopping the use of triptolide, male fertility recovery was slow, indicating that triptolide not only destroys germ cells in the testes but also damages epididymal sperm. Data analysis show that the potential mechanism of reproductive toxicity induced by triptolide may involve the interference of genes related to spermatogenesis.

## 6. Derivatives of triptolide

**Figure 6:**
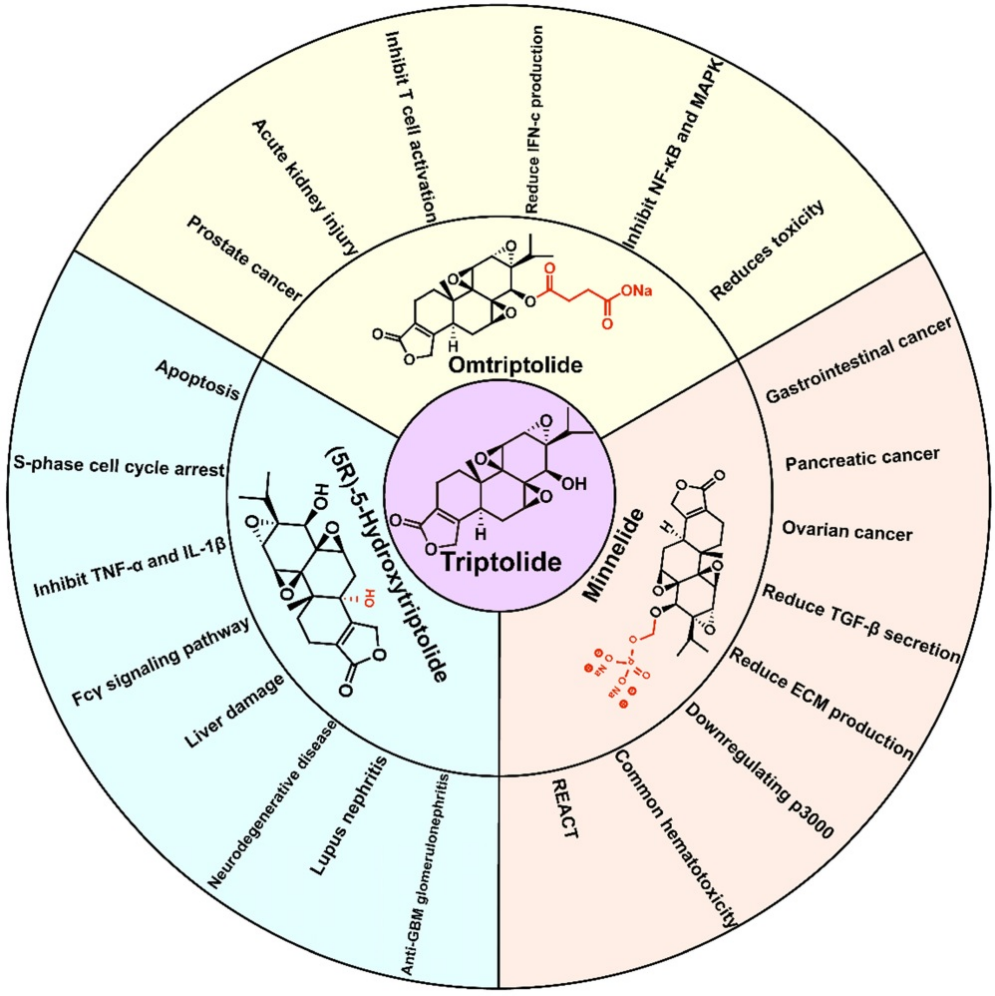
Common triptolide derivatives and their research contents.

Although triptolide has strong pharmacological activity, its clinical application is severely restricted due to its poor solubility and bioavailability, and the serious toxicity and side effects it induces, and a narrow therapeutic window. In recent years, researchers have modified the structure of triptolide to increase its water solubility and reduce the toxicity and side effects it induces without affecting its activity. Currently, a variety of triptolide derivatives, such as omtriptolide and minnelide (Figure 6) have been investigated, and some have entered the clinical trial stage (Table 2).

### 6.1. Omtriptolide

**Scheme 5:**
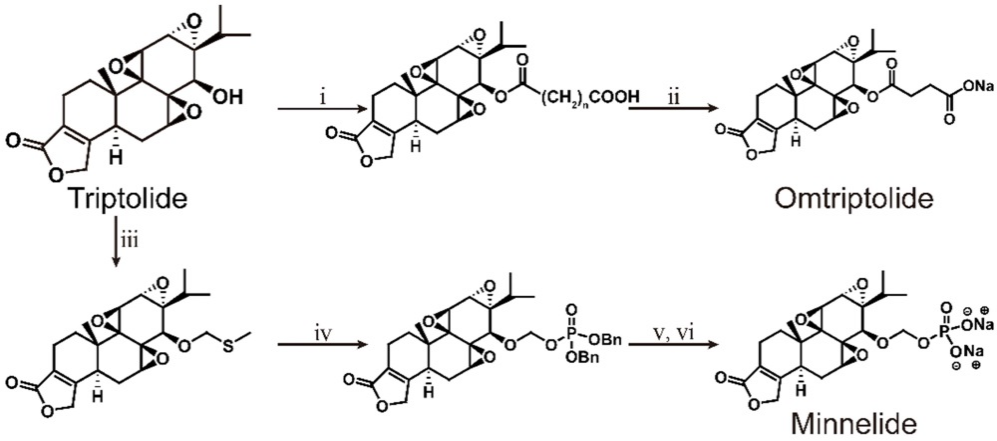
Total synthesis of Omtriptolide and Minnelide. ⅰ) HO_2_C(CH_2_)_n_CO_2_H, DCC/DMAP; ⅱ) n=2; ⅲ) Me_2_S, MeCN, BPO, 2 h; ⅳ) CH_2_Cl_2_, 4A MS, (BnO)_2_P(O)OH, NIS, THF; ⅴ) H_2_, Pb/C, THF; ⅵ) THF, Na_2_CO_3_.

As early as 1997, researchers designed a new semisynthetic water-soluble derivative, omtriptolide (PG490-88, f60008), by introducing a fatty acid structure into triptolide C-14 140. Omtriptolide shows immunosuppressive and antitumor activities (scheme 5), and it is currently approved for phase I clinical trials of prostate cancer in the United States 141. Long-term research results show that PG490-88 exhibits cytotoxicity in tumor cell lines, including H23 (NSCLC), HT1080 (fibrosarcoma) and COLO 205 (colon cancer) cells 4. Studies have shown that when PG490-88 is used alone, it can cause the regression of lung cancer and colon cancer xenograft tumors, and the synergistic effect of PG490-88 and CPT-11 can also cause tumor regression 142.

In recent years, it has been found that PG490-88 can reduce the disease progression of kidney disease in various animal models. PG490-88 and tacrolimus (Tac) work synergistically to inhibit T cell activation and reduce IFN-c production and NF-AT/NF-jB activity, thereby prolonging the survival time of transplanted kidneys in a monkey model 143. Some scholars have found that PG490-88 can attenuate acute humoural rejection by inhibiting complement activation and T cell infiltration, thereby significantly prolonging the survival time dog models after kidney transplantation 144. Moreover, PG490-88 may reduce the amount of p-ERK released in cisplatin-induced acute kidney injury (AKI), thereby protecting against AKI and acute tubular necrosis (ATN) 145. The latest research confirmed that PG490-88 can inhibit the activation of the NF-κB and MAPK signaling pathways as well as the production of pro-inflammatory mediators and cytokines and ultimately plays a protective role in I/R-injured rats 146.

Researchers conducted a phase I and pharmacological study of PG490-88 in patients with advanced solid tumors 141. The adverse reactions were mainly fatigue, nausea, vomiting, diarrhoea, and constipation. The haematological side effects were mild grade 1 anaemia, but no liver or kidney toxicity was found. However, in two cases, the side effects were fatal. One patient died of neutrophilic sepsis, and another patient may have died of a complex clinical syndrome caused by cytokine release. Ultimately, researchers believe that the degree of PG490-88 conversion to triptolide in the human body is unpredictable; therefore, PG490-88 is not the best derivative of triptolide to use in the clinic. Phase I clinical trials were forced to be discontinued in 2009. According to the current experimental results, PG490-88 has a strong anticancer effect and reduced liver and kidney toxicity compared to triptolide, which provides a reference for the clinical application of triptolide.

### 6.2. (5R)-5-Hydroxytriptolide

**Scheme 6:**
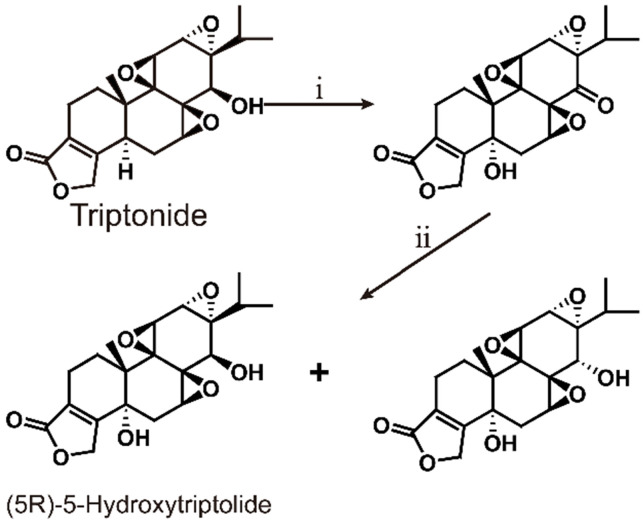
Total synthesis of (5R)-5-Hydroxytriptolide. ⅰ) SeO_2_, heat reflux 10 h; ⅱ) NaBH_4_, 2 h

(5R)-5-Hydroxytriptolide (LLDT-8) is a new triptolide analogue with strong immunosuppressive and anti-inflammatory activity 147 (Scheme 6). Currently, LLDT-8 is being investigated as a low-toxicity immunosuppressive agent in Phase I clinical trials for the treatment of RA in China 148, and the results show that LLDT-8 can inhibit the production of MMP-13 and increase the expression of OPG/RANKL through the OPG/RANK/RANK ligand signaling pathway to weaken collagen-induced arthritis (CIA). This experiment used CIA to simulate RA 149. Early studies have shown that LLDT-8 may be closely related to anti-inflammatory, antioxidant and cytokine effects and can protect against bleomycin-induced lung fibrosis in mice 150. Recently, researchers constructed the first lncRNA-TF-mRNA coexpression network, which further explain changes in whole-genome lncRNA and mRNA expression before and after LLDT-8 treatment. The authors believe that lncRNAs may be biomarkers and targets for LLDT-8 drug development 151. At the same time, LLDT-8 showed a strong anti-inflammatory effect, for example, by regulating the Fcγ signaling pathway to improve anti-GBM glomerulonephritis 152 and inhibiting the expression of renal chemokines to suppress infiltration of kidney immune cells, thereby improving lupus nephritis 153.

LLDT-8 also has a certain therapeutic effect on neurological diseases. Some scholars have studied the anti-inflammatory and neuroprotective effects of LLDT-8 on cerebral ischaemia-reperfusion injury. The results show that it may inhibit the neuroinflammation mediated by microglia through the IκB/NF-κB cascade, play an anti-inflammatory effect, and protect against acute cerebral ischaemia-reperfusion injury 154. Studies have shown that LLDT-8 can reduce PD-like behaviour and dopaminergic neurodegeneration and neuroinflammation of the nigrostriatal system, providing a new method and entry point for the treatment of PD 155. LLDT-8 can effectively inhibit pro-inflammatory factors (TNF-α and IL-1β) to suppress the NF-κB signaling pathway and thereby reduce neuroinflammation, and it is used to treat neurodegenerative diseases 156. In addition, LLDT-8 can also reduce serum alanine aminotransferase (ALT) and aspartate aminotransferase (AST) levels and reduce liver balloon cell formation and macrocystic steatosis, thereby inhibiting liver damage 157. LLDT-8 can also regulate the expression levels of stearoyl-CoA desaturase 1 (SCD1) and hepatic peroxisome proliferator-activated receptor α (PPARα), significantly promoting lipid breakdown and inhibiting lipid synthesis.

Clinical trials confirmed that LLDT-8 has broad-spectrum antitumor activity, inducing S-phase cell cycle arrest and apoptosis. As a novel transcription inhibitor, LLDT-8 has a potential therapeutic effect on P-glycoprotein-mediated drug-resistant tumors 158. Researchers used mouse spleen cells for LLDT-8 cytotoxicity experiments and found that although the inhibitory activity of LLDT-8 against ConA and LPS cell proliferation was reduced 20-fold and the inhibitory activity against allo-MLR was reduced 14-fold, the toxicity of LLDT-8 was reduced 122-fold compared with triptolide. In addition, data from an acute toxicity study showed that LLDT-8 exhibited a 10-fold reduction in toxicity in mice 159. Liquid chromatography-tandem mass spectrometry (LC-MS/MS) used for the analysis and characterization and benzylamine chemical derivatization to determine the content of LLDT-8 in human plasma is a more sensitive and reliable quantitative method for studying pharmacokinetics 148. This method covers a wide linear dynamic range (0.030-100 ng/ml), and within this linear range, it shows better accuracy (RE<11.7) and precision (RSD<8.6). Considering current clinical research, LLDT-8 may be a more suitable clinical alternative drug for triptolide. In terms of pharmacological activity, LLDT-8 not only retains the strong immunosuppressive and anti-inflammatory activity of triptolide but also has broad-spectrum antitumor activity. In clinical applications, compared with triptolide, the toxicity of LLDT-8 is greatly reduced.

### 6.3. Minnelide

Minnelide is a water-soluble derivative formed by adding a phosphate group to triptolide, endowing it with a wide range of anticancer effects (Scheme 5). Currently, minnelide has entered Phase I clinical trials for gastrointestinal cancer and pancreatic cancer 160, 161. Studies have shown that minnelide can eliminate colon cancer cells *in vitro*, reduce the growth of primary colon cancer, and metastasize colorectal cancer to the liver *in vivo* and may become a new strategy for the treatment of primary and metastatic colon cancer 162. A University of Minnesota team found that minnelide reduces CD133+-derived tumor volume and the number of tumor-initiating cells (TICs) in the tumor 163. This is the first report on the efficacy of minnelide in the syngeneic system of immune tolerance. Subsequently, the group conducted in-depth research and established an *in vitro* model for studying the characteristics of tumor stem cells and tumor-initiating cells and found that minnelide has good prospects in a preclinical evaluation 164. The team also found that minnelide can deplete extracellular matrix components by depleting hyaluronic acid and collagen to improve drug delivery and survival 165.

In addition to pancreatic cancer and gastrointestinal cancer, minnelide can also inhibit the activity of NF-κB and prevent metastasis. It effectively reduces the tumor burden and metastasis potential of osteosarcoma, has the least impact on osteoblasts of the tested compounds, and may be developed into a very effective chemotherapy drug for osteosarcoma 166. Studies have confirmed that minnelide can induce cell death of prostate cancer cells by downregulating AR and its variants 167. Minnelide can effectively inhibit the proliferation of platinum-sensitive and drug-resistant ovarian cancer cell lines, thereby improving the efficacy of standard chemotherapy, such as carboplatin and paclitaxel 160, 168. In addition, minnelide combined with an anti-DR5 monoclonal antibody provides a new therapeutic option against metastatic renal cell carcinoma 169.

In recent years, minnelide has mainly been investigated for the treatment of pancreatic cancer and has entered a phase Ⅱ clinical trial of advanced pancreatic cancer 170. Minnelide is the fastest developed derivative of triptolide. Clinical trials show that the maximum patient-tolerated dose is 0.2 mg/kg. At this dose, minnelide can induce irreversible CAFs to acquire an inactive state phenotype and reduce TGF-β secretion and ECM production, thereby reducing the proliferation of TECs to cause tumor regression 171. Minnelide can also inhibit the pro-survival signaling in pancreatic cancer cells by downregulating the expression of p3000 and reducing the transcriptional activity of the HIF-1α transcription complex. Furthermore, minnelide regulates downstream effects by reducing hypoxia and associated signaling 172.

The results of a phase I clinical trial in 27 patients with refractory gastrointestinal cancer (17 pancreas, 7 large intestine and 3 other gastrointestinal tract cancers) showed that the overall treatment effect was good except for haematotoxicity that common among the patients and usually was resolved within 2-3 days after drug withdrawal 173. Minnelide was administered by intravenous infusion every 28 days on days 1-5, 8-12, and 15-19, with a dose range of 0.16-0.8 mg/m^2^. However, two patients may have suffered reversible acute cerebellar toxicity (REACT) in a phase I clinical trial of minnelide. The patient's imaging scans showed a slight increase in the PLAIR signal and a marked decrease in cerebellar cortical spread 174. However, the imaging-based findings of another patient indicated a nearly opposite situation: They revealed possible post-reversible encephalopathy syndrome or acute toxic leukoencephalopathy. These results indicate that minnelide may cause potential reversible acute cerebellar toxicity. Therefore, more in-depth research and analysis are needed in the course of clinical trials. The latest research shows that c-myc transcription in AML cells can be inhibited in a range far lower than the equivalent human dose that patients can safely tolerate in phase I clinical trials, which leads to cell cycle arrest and apoptosis, thus significantly reducing the burden of leukaemia 175. After entering the body, minnelide can be rapidly and completely converted into triptolide, which is beneficial for controlling the dosage of the drug, improving its safety and effectiveness.

## 7. Conclusions and prospects

Triptolide is the main active ingredient of the traditional Chinese medicinal herb *T. wilfordii*. It has a unique chemical structure and promising pharmacological activity. But there are still many challenges in the translation or triptolide from use in traditional to modern drugs, such as poor water solubility, a narrow treatment window, and strong toxicity and side effects. At this stage, the main solution is to modify the different parts of the triptolide structure, such as C-14 hydroxyl group, to obtain derivatives that have improved poor water solubility and fewer and less severe side effects.

In addition, the production of triptolide is also a major challenge. The traditional method for obtaining triptolide cannot meet commercial needs. With good results, research on the total synthesis of triptolide has led to researchers analyzing and optimizing synthetic routes of triptolide. At present, the research on the chemical synthesis of triptolide mainly focuses on the optimization and innovation of synthesis conditions, so as to improve the yield of triptolide. In addition, the use of biosynthetic methods to produce triptolide is the research focus of many groups. Although the biosynthetic pathway of triptolide has not been fully elucidated, the upstream biosynthetic pathway of terpenoids in *T. wilfordii* has been analyzed. Moreover, high-quality genome sequencing and annotation of *T. wilfordii* genes have been completed, laying the foundation for better identification of genes encoding key enzymes in the triptolide biosynthesis pathway.

Many reviews have been focused on triptolide. However, thus far, these reviews have been basically aimed at introducing the research progress of triptolide, such as its anticancer effects 176, derivative identification 170 and advances in its total chemical synthesis 119. In this review, triptolide is described in detail from different perspectives such as pharmacological activity and biosynthesis, aiming to provide new ideas for researchers in different disciplines and promote the research progress of triptolide. Currently, the biosynthetic pathway of triptolide has been resolved sufficiently to identify dehydroabietic acid as a key intermediate, and the chemical synthesis of triptolide from dehydroabietic acid has been reported by scholars. This progress suggests that, in the near future research, researchers can combine biosynthesis with chemical synthesis to realize the industrial production of triptolide. Similarly, the combination of pharmacological activity research and synthetic pathway analysis can be used to study the pharmacological activities of intermediates similar to triptolide through synthetic pathways to screen analogues with the rich pharmacological activities of triptolide that induce fewer side effects, such as triptinin B and triptophenolide. For the research of triptolide derivatives, the common derivatization methods include hydroxylation or glycosylation, which may be realized by cytochrome P450 and glycosyltransferase. The analysis of biosynthetic pathways also includes dioxygenase and methyltransferase, which provide more possibilities for the study of triptolide derivatives. In the study of the pharmacological activities of triptolide derivatives and triptolide, many pathways and targets are the same, which can provide more ideas for improving the production of triptolide and its derivatives.

## Figures and Tables

**Table 1:** Anticancer effect of triptolide on different cancers.

Tumor model	Pathway	Mechanism	Cell or animal model	Refs
**Lung cancer**	RPL23-MDM2-p53	Induce apoptosis, cell cycle arrest and inhibition of cell proliferation	Lung cancer A549 cells (CCL‑185™) BALB/cAnNCr‑nu/nu mice	28
	Micro-RNA	Reduce cancer cell migration and invasion	H460, A549, and H358 cells Nondiabetic severe combined immune deficiency γ mice	29
	PI3K/AKT/mTOR	Induce apoptosis, inhibition of cell proliferation, glycolysis and energy metabolism	H1299 and NCI-H460 cell	30
	Nrf2-ARE	Enhance cancer cell sensitivity to chemotherapy drugs	Non-small-cell lung cancer (A549) Mouse lung carcinoma 3LL cell liver carcinoma (HepG2) cell	32
**Liver cancer**	c-Myc/micro-RNA	Induce apoptosis	Liver cancer cell lines SMMC-7721, MHCC-97H, and LM3 HepG2 and Hep3B	35
	gene p53	Inhibit the vitality of cancer cells Induce apoptosis	HepG2 and QSG7701 liver cancer cell	36
**Nerve tumor**	ROS/JNK Akt/mTOR	Induced G2/M phase arrest, apoptosis, and autophagy	U251, U87-MG and C6 cells BALB/c-nu/nu nude mice	37
	IFN-γ	Inhibits PD-L1 expression Reversing the inhibition of CD4+ T cells by cancer cells	Glioma cell lines, U251-MG, T98G, U87-MG, A172, LN229 and LN18	38
	MicroRNA‐138 PI3K/AKT Notch	Induces apoptosis and inhibits cancer cell migration and proliferation	Human medulloblastoma Daoy cells and human embryonic kidney HEK293 cells	39
	MicroRNA-181a p38MAPK NF-kB	Inhibit the proliferation and migration of cancer cells	Human neuroblastoma SH-SY5Y cells	40
**Pancreatic cancer**	b-Catenin/Wnt	Induces apoptosis of cancer cells	The pancreatic cancer cell line MIA PaCa-2 The S2-VP10 cell lines The BxPC-3, Capan-1 and Human pancreatic ductal epithelial cells Genetically engineered KPC (Kras^G12D^, P53^R172H^PDX^Cre^) mice	177
	TLR4/NF-κB	Enhances the sensitivity of pancreatic cancer PANC-1 cells to GEM	The human pancreatic cancer cell line PANC-1 Balb/c nude mice	42
	TGF-β/Smad	Modulate tumor microenvironment Reduces extracellular matrix (ecm) production	The human pancreatic cancer cell line PANC-1 Mouse embryonic fibroblast cell line NIH3T3 The human pancreatic cancer cell PANC-1-Luc2 Pancreatic tumor-bearing nude mice	45
**Prostate cancer**	Caveolin-1/CD147/MMPs	Inhibition of cancer cell migration and invasion	PC-3 and DU145 human prostate cancer cell	46

**Table 2:** Related research on triptolide and its derivatives.

Compound	Chemical structure	Aqueous solubility	Pharmacological activity	Refs
Triptolide (PG490, LDTT-2)	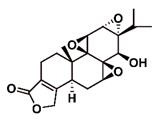	Poor Solubility	Anti-cancer, anti-inflammatory and immunosuppression	22, 23 4
Omtriptolide (PG490-88, F60008)	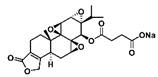	Soluble	Anti-cancer, immunosuppression, inhibition of pro-inflammatory mediators and cytokines	4 146
(5R)-5-Hydroxytriptolide (LLDT-8)	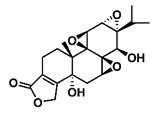	Soluble	Immunosuppression and anti-inflammatory effects	151, 155
Minnelide	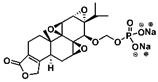	Soluble	Extensive anti-cancer effects enhance the efficacy of chemotherapy drugs	160, 168
